# Progression from being at-risk to psychosis: next steps

**DOI:** 10.1038/s41537-020-00117-0

**Published:** 2020-10-05

**Authors:** Jean Addington, Megan Farris, Daniel Devoe, Paul Metzak

**Affiliations:** Hotchkiss Brain Institute, Department of Psychiatry, University of Calgary, Calgary, AB Canada

**Keywords:** Human behaviour, Psychosis

## Abstract

Over the past 20 years there has been a great deal of research into those considered to be at risk for developing psychosis. Much has been learned and studies have been encouraging. The aim of this paper is to offer an update of the current status of research on risk for psychosis, and what the next steps might be in examining the progression from CHR to psychosis. Advances have been made in accurate prediction, yet there are some methodological issues in ascertainment, diagnosis, the use of data-driven selection methods and lack of external validation. Although there have been several high-quality treatment trials the heterogeneity of this clinical high-risk population has to be addressed so that their treatment needs can be properly met. Recommendations for the future include more collaborative research programmes, and ensuring they are accessible and harmonized with respect to criteria and outcomes so that the field can continue to move forward with the development of large collaborative consortiums as well as increased funding for multisite projects.

## Introduction

In the past two decades researchers have attempted to identify and assess individuals who are at risk for psychosis, with the hope that this could lead to substantive improvements for the outcome of serious mental illnesses such as schizophrenia. The criteria for identifying these young people is mainly based on attenuated psychotic symptoms (APS), which were originally developed as suggestive of being putatively prodromal for psychosis. However, since prodrome is a retrospective concept, that is a prodrome for an illness only exists once that specific illness has occurred, and secondly, because the majority of those who are at risk for psychosis do not go on to develop a full-blown psychotic illness, prodrome should be reserved to retrospectively describe the at-risk period for those who have developed a psychotic illness. The terminology used to describe these young people are being at clinical high-risk (CHR) or at ultra-high risk (UHR) for psychosis. In this paper for consistency we will use CHR.

There are well developed criteria for CHR and UHR based on structured clinical interviews; the two most common being the Scale of Psychosis-Risk Syndromes (SIPS^1^), and the Comprehensive Assessment of At-Risk Mental States (CAARMS^2^). A meta-analysis published in 2011 describing rates of transition to psychosis suggested that for those identified as being at CHR, ~20–35% will develop psychosis within 2 years^3^. Furthermore, the risk of developing psychosis in these young people is often imminent, as most transitions occur within the first year after study ascertainment; thereafter the risk of transition decelerates^4^. The initial key goals of high risk for psychosis research were to identify those at risk of psychosis earlier, to identify and refine predictors of transition to psychosis beyond the reported 20–35%, and finally to develop treatments that can prevent psychosis. However, recent research^5^ demonstrates that although the majority of CHR individuals do not go on to develop psychosis they often continue to experience APS, and have poor functioning as well as many other comorbid problems^6^. Thus, an additional worthy goal is an improved understanding of those who do not transition to psychosis and designing relevant treatments for these young people.

**Fig. 1:**
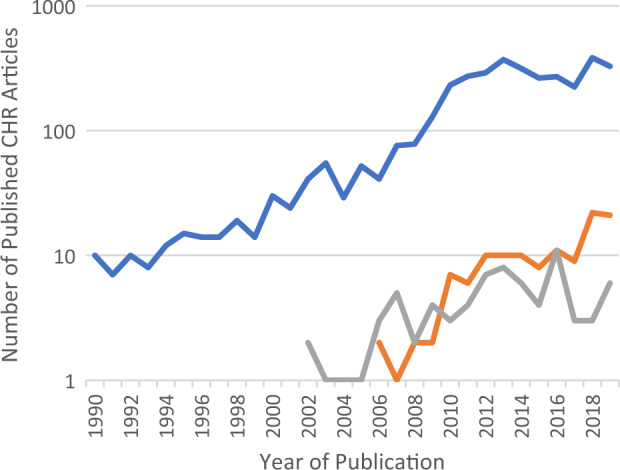
Types of CHR Articles Stratified by Year. The blue line indicates any CHR article; the orange line indicates review papers; andthe grey line indicates CHR treatment studies.

We conducted an informal electronic database search of article titles and abstracts to gain an approximation of the current state of CHR research and to examine the incidence of treatment publications versus other research publications versus meta-analyses and reviews stratified by time (Fig. 1). Clinical high-risk nontreatment research has increased exponentially over the last decade growing from an average of nine studies per year in 1990–1994 to an average of 300 articles per year in 2014–2019. In comparison, the number of CHR treatment articles published in the same timeframe was much less, and remains relatively low, and are currently being surpassed by reviews that vary in quality. Despite the rarity of CHR and psychosis, with the increased number of publications from multiple research programs and clinics worldwide (including multisite international studies), huge numbers of young people meeting established criteria for CHR are being identified and studied. Therefore, the aim of this paper is to offer an update of the current status of research on risk for psychosis, and what the next steps might be for examining the progression from CHR to psychosis. Updates on prediction and treatment studies will first be presented, followed by a consideration of the issue of heterogeneity in CHR and recommendations for future directions in this field.

## Predictors of transition to psychosis

Literature in this area focuses on individual risk factors and more recently, prediction models of risk considering several risk factors. There have been three recent reviews^7–9^ that have summarized both individual risk factors as well as prediction models. These reviews present solid support for the role of clinical factors such as: poor cognition, poor social functioning and a decline in social functioning as predictors of transition to psychosis; as well as certain environmental factors such as: trauma, bullying, and cannabis use^10–13^. Research is beginning to examine the possible role of certain neurobiological changes in the transition to psychosis. However, it should be noted that although some neurobiological changes suggest that there are changes occurring during the high risk and/or transition stage and that there are observed differences between those who do and those who do not transition to psychosis, these changes may not yet be strong enough to predict transition.

Longitudinal structural magnetic resonance imaging (MRI) findings suggest that an increased rate of grey matter loss, particularly in the frontal lobes, may be a predictor of transition to psychosis in CHR individuals^14,15^, which is supported by other longitudinal studies employing machine learning^16,17^. Several functional MRI (fMRI) studies have suggested that transition to psychosis might be associated with cerebello-thalamo-cortical hyperconnectivity^18,19^ or other bidirectional changes in thalamic connectivity^20^. Although these findings are promising, much more work is required to characterize and understand these changes in brain structure and function. Further, although many of these reported changes may be related to transition, at present, none of the neuroimaging findings can be used to identify CHR individuals who will transition. The neurobiological changes that precede transition to psychosis appear to be subtle and there is a high degree of heterogeneity in the findings. This heterogeneity is likely the consequence of many factors, including but not limited to, variability in individuals at CHR (as discussed in a later section), as well as factors related to experimental design and differences in analysis strategies.

Neurobiological changes in CHR individuals have also been detected using neurophysiology, in particular electroencephalogram (EEG) studies. Some of the more promising EEG parameters that might be related to transition to psychosis have been reductions in mismatch negativity^9,21^, and the P300 event-related potential responses^22,23^.

There are, however, far fewer studies examining potentially important serologic changes such as blood biomarkers, neuroinflammation and stress hormones^9^. There is some preliminary evidence that elevated baseline plasma levels of particular inflammation markers, oxidative stress and dysregulation of the hypothalamic–pituitary–adrenal axis might be associated with transition to psychosis^24,25^. Furthermore, although CHR individuals may have elevated cortisol, it does not appear to be related to transition^26^. Finally, there is very early evidence implicating prolactin and the hypothalamic–pituitary-gonadal axis^27^, and accumulating evidence that the polygenic risk score may be a factor in the later development of psychosis^28^.

### Models of prediction

The next step in prediction is to focus on developing models^7,8^. As the most common clinical predictors seem to be cognition and functioning, several studies have increased their predictive value by including aspects of cognition, functioning and/or negative symptoms^29–33^. A key study in combining clinical and biomarkers demonstrated that the combination of age and gender, family history of psychosis, symptoms, blood biomarkers and EEG measures identified over 70% of CHR participants who made the transition to psychosis in one year versus the 28% by the standard CHR criteria^34^. Although a small study this is one of the first to combine clinical and biomarker data.

An individualized risk calculator, comparable in accuracy to those used for cancer and cardiovascular disease^35^, was developed through a large CHR project, the North American Prodrome Longitudinal Study (NAPLS). This calculator only included variables that had been supported by earlier studies and could be accessed in a clinical setting. The predictors that were found to contribute to an individual’s risk of transition to psychosis included increased ratings on unusual thought content and suspiciousness, greater decline in social functioning, poorer verbal learning, memory and speed of processing and younger age. This model had a concordance index of 0.71^35^. The calculator was externally validated^36^ demonstrating good discrimination with an AUC of 79%, a sensitivity of 91% and a specificity of 37% compared to 94.1% and 23.6% in the NAPLS sample. The concordance index of 0.71 is in the range of values for established calculators currently in use for cardiovascular disease and cancer recurrence risk, which range from 0.58–0.81^37–40^. The calculator is available as a web-based tool (http://riskcalc.org:3838/napls/). However, this risk calculator is only valid for predicting transition to psychosis risk in those who meet criteria for being at CHR based on the SIPS, which suggests that it is most appropriate for use in research including clinical trials.

### Current issues with prediction

The recent reviews^7–9^ identified several methodological issues that potentially impact prediction including different methods of ascertainment of CHR individuals, different CHR criteria, a wide range of assessment batteries, model over-fitting, using data-driven variable selection methods rather than the scientific literature and lack of external validation^9,36,41,42^. However, some of these concerns have been preliminarily addressed in recent studies. For example, it was recommended to select candidate predictors based on scientific literature versus the variables from a given study’s test battery^43^. Further, more advanced statistical methods should be considered to enhance predictive accuracy in these models, such as the least absolute shrinkage and selection operator(LASSO), ridge regression or elastic net techniques. Finally, since prediction algorithms typically use only baseline predictors, dynamic prediction, which involves longitudinal data collection of the predictors at subsequent assessment(s), is a potential option^44,45^.

Since the 2019 paper reviewing psychosis-risk prediction models in CHR^7^, there have been three articles published on the subject. The first supported the joint modelling approach by developing a dynamic risk prediction model which used longitudinal data to better understand psychosis risk^46^. The authors found that a dynamic prediction model could be implemented in CHR and resulted in significantly better sensitivity, specificity and likelihood ratios, relative to other predictive risk models using static (baseline) variables. Thus, this dynamic model appeared to be a better method for prediction of transition to psychosis in CHR. The second paper is one of the first and largest meta-analysis of both risk and protective factors that might predict transition^47^. Reviewing 128 studies and 26 potential risk factors, the authors identified the most robust risk factors for psychosis against different types of biases^48^, classifying the levels of evidence of the association as convincing (Class I), highly suggestive (Class II), suggestive (Class III) and not met or weak (Class IV). No factors showed Class I-convincing evidence, but APS and global functioning evidence were associated with Class II evidence. There was Class III evidence for negative symptoms. Although not novel, these findings provide important support to some of the work presented earlier.

A third paper^49^ describes the Psychosis Polyrisk Score (PPS), which was developed to measure the multivariable exposure to several risk factors that could contribute to the development of psychosis. Polygenetic risk scores^28^ have recently been used to examine prediction, but the use of non-genetic factors has been more limited. Leveraging Class I and II risk factors, the PPS was developed incorporating robust epidemiological risk factors for psychosis.

It is possible that prediction of transition to psychosis will be improved by incorporating not only clinical factors, but also factors from domains that might contribute to a greater understanding of the interaction between clinical, environmental and neurobiological factors^9^. These domains may include neuroimaging, electrophysiology or serology, either individually or in combination.

## Treatment

Based on the comprehensive reviews^50^ and meta-analyses discussed below, over 50 treatment studies have been conducted in the CHR literature. However, in addition to the 20 randomized controlled trials (RCTs) examining the impact of treatment for individuals at CHR, there are also small pilot studies, open trials and trials that recruit mixed populations, such as those that include both CHR individuals and first episode psychosis patients. In these trials, treatment modalities are highly variable and include cognitive behavior therapy (CBT), cognitive remediation, family interventions, integrative psychological therapy, antipsychotics, omega-3 fatty acids, d-serine and glycine^50^. The most common outcomes were transition to a psychotic disorder, APS and global functioning. We have previously reviewed in detail the key RCTs where 10 used a psychosocial treatment, seven were pharmacological and three used a combination treatment^50^. We present a brief summary below.

The most common and probably most successful intervention to date is CBT. In both the EDIE and EDIE-2 trials^51,52^ CBT was compared to monitoring. The EDIE trial reported, for the CBT group, a 96% reduction in the odds of making a transition to psychosis and a reduction in the severity of APS. Although in EDIE-2 there was no impact on transition, the frequency and intensity of APS for those receiving CBT was significantly reduced. Interestingly, the overall transition rate in EDIE-2 was less than 8% within 2 years; an issue that was later observed in some other CHR treatment studies. The Dutch EDIE trial^53,54^ reported that compared to monitoring, transition was reduced by 50% in their CBT group, an outcome that was still significant at a 4-year follow-up. In the two RCTs that compared an active treatment to CBT, one did not show any transition or symptom differences between supportive therapy and CBT^55^ and the other demonstrated that the Non-Directive Reflective Listening condition was superior to the CBT condition in decreasing the distress related to attenuated psychotic symptoms^56^. However, in these studies both groups demonstrated improvement and again, transition rates were low.

Only one RCT used a family intervention comparing three sessions of psychoeducation on stress management to Family Focused Therapy (FFT), which was an 18-session therapy of symptom management, communication, social and problem-solving^57^. Those receiving FFT had reduced APS and family conflict and improved communication. Although there were no differences in transition rates, improvement in negative symptoms and social and role functioning was observed in both groups. Similarly, only one RCT to date has utilized an Integrative Psychological Therapy (IPT) design in CHR^58^. Consisting of CBT, skills training, cognitive remediation, and a psychoeducational multifamily group, IPT significantly reduced transition to psychosis at both 12- and 24-month follow-up compared to supportive counselling.

Results of two RCTs comparing N-methyl-D-aspartate receptor modulators (D-serine or glycine) to placebo reported that there no effect of glycine on any measure^59^, however, the use of D-serine study demonstrated a significant improvement in negative symptoms compared to placebo^60^. Mixed results have emerged from trials of antipsychotics both with and without psychosocial treatments^61–65^. Generally antipsychotics failed to reduce transition but did improve APS. Similarly, there have been mixed results from the three RCTs comparing omega-3 fatty acids to placebo^66–68^.

### Systematic reviews of treatment

Numerous recently published meta-analyses have attempted to synthesise treatment efficacy on a diverse set of outcomes including transition to a psychotic disorder^69,70^, social functioning^71^, attenuated psychotic symptoms^72,73^ and negative symptoms^74^. Reviews have examined the treatment evidence using both conventional pairwise meta-analysis and network meta-analysis (NMA). An NMA allows for evaluations between multiple treatments (i.e. three or more) in CHR for psychosis trials using direct and indirect comparisons of interventions within and across RCTs based on a common comparator (such as needs-based interventions or placebo). Although NMAs have several advantages, one such advantage being the ability to compare interventions that have not been directly compared in an RCT, it is recommended that researchers report and compare the results from both pairwise and NMAs for a comprehensive understanding of the literature^75^.

Two recent NMAs examined transition to psychosis^69,70^, two more examined the impact of RCTs on APS^72,73^, and a fifth negative symptoms^74^. None of these NMAs were able to show that any one treatment was more effective in reducing transition, APS or negative symptoms. This does not mean that the treatments had no effect, it means is that none of the treatments differed from one another in terms of effectiveness. This may be due to several factors. In all these NMAs, the analyses typically included very few trials and often treatment comparisons were represented by a single trial. Thus, the networks had sparse connections, which inevitably led to imprecise estimates and wide confidence intervals. Results of these NMAs may significantly change as more evidence emerges from future RCTs.

However, there have been several pairwise meta-analyses published either in conjunction with the NMA or independently. In the pairwise meta-analysis by Devoe et al.^70^, comparing CBT to relevant comparators, CBT was associated with a statistically significant reduction in transition to psychosis at 12- and 18-months. The main limitation of this pairwise meta-analysis was the lack of consistency in the comparison conditions (e.g. supportive therapy, needs-based interventions, etc.). However, the conclusion that CBT was superior at preventing transition to psychosis is informative as the results were based exclusively on direct evidence. CBT was also associated with trend-level reductions in attenuated psychotic symptoms compared to control treatment at 12 months^72^. There was, however, no evidence for any one treatment being effective in reducing negative symptoms^74^, or social functioning in CHR individuals^71^. The limitations of these analyses were that most RCTs were not designed to target negative symptoms or functioning as primary outcomes.

In the period after these reviews were published, a Cochrane Review^76^ was added to the literature which concluded that “the evidence available suggests that omega‐3 fatty acids may prevent transition to psychosis” and that “more research is needed to confirm this finding”. However, this Cochrane Review had some methodological flaws as the last search they conducted was in August 2017 and subsequently published the review 2 years later. The most concerning element of the Cochrane Review is that the largest Omega-3 study (i.e. NEURAPRO study)^68^, published in JAMA psychiatry in January 2017, was missed even though it was published within the Cochrane Review’s search dates. This study included 304 participants and concluded that Omega-3 clearly failed to replicate the findings of the first trial, and that Omega-3 was not effective at preventing transition relative to placebo. Furthermore, at least one meta-analyses published before the Cochrane Review, included three Omega-3 trials and had already concluded that there is no aggregate evidence that Omega-3 reduces transition at any timepoint^77^. A more detailed criticism of this review addressing its methodological concerns has been published by Nelson et al. in the Lancet Psychiatry^78^.

As a further update to the literature, a recent search in SCOPUS for trials published between July 2017 and October 2019 was conducted and revealed three relevant RCTs examining the efficacy of: cannabidiol^79^, oxytocin^80^ and systemic therapy^81^ in CHR individuals. The trial examining systemic therapy showed a trend towards significant improvements in positive and depressive symptoms as well as social support and self-esteem, relative to supportive therapy^81^. The cannabidiol RCT^79^ found that it may partially normalize alterations in parahipppocampal, striatal and midbrain function linked with the CHR state. Finally, the oxytocin RCT^80^ found that, relative to placebo, the administration of oxytocin was associated with increased hippocampal blood flow, although the effect at the second timepoint was not maintained after adjustment for the effect of global blood flow.

### What does this mean for treatment?

Overall, it is the psychosocial treatments, in particular CBT, that have demonstrated significant improvements in CHR individuals relative to monitoring or placebo. Several issues may have impacted the results of these trials including some methodological concerns. First, even with the recent additions, the current number of RCTs does not exceed 25, with very few studies for each treatment modality conducted over a wide time span (~20 years since the first study in Melbourne was published)^62^. Second, the outcome in most of these trials was transition to psychosis, yet the rates of transition have been declining overtime from ~35% in the earlier studies to 8–11% in more recent studies, reasons for which are unclear^82^. Third, APS do decline in severity overtime plus some CHR individuals are in remission from APS within the first few months^5,83^. Fourth, many of the studies report multiple outcomes, even those for which the treatment was unlikely to have been designed. For example, most trials presented functioning as an outcome, yet none of the trials reviewed were designed specifically to improve functioning. Finally, heterogeneity in CHR samples is another critical confound in these studies, as will be discussed later in this article.

However, what must be emphasized is that it is possible that any kind of treatment might be helpful for CHR youth, as trial participants did show improvements particularly in clinical symptoms, even though there may not have been significant differences between the treatments being compared. Furthermore, potential differences may have been obscured if participants were receiving other treatments, such as medication or support, supplementary to treatment not under experimental investigation. For example, in the omega-3 RCT^68^, both omega-3 and placebo groups received cognitive-behavioural case management (CBCM) and possibly antidepressants, which may have limited the measurable efficacy of omega-3^84^.

## Clinical heterogeneity

One issue that has been raised several times is the heterogenous presentation of CHR samples. This clinical heterogeneity has likely played a role in the outcomes of many clinical trials. Heterogeneity is first observed at the diagnostic level^85^. In addition to attenuated psychotic symptoms, these young people often suffer from comorbidities, in particular anxiety and depression^86–88^. Poor social and role functioning is common and is at times, equivalent to those experiencing a first episode of psychosis^10^. Many, but not all, have negative symptoms^89–91^ and in some cases, these negative symptoms endure long enough that they are identified as being persistent negative symptoms^92^. As a group, CHR individuals have problems with neurocognition, which tends to be intermediary to healthy controls and those with a first episode of psychosis^93,94^. Cannabis use is also of concern in this population and a recent review suggests that ~50% of CHR individuals use cannabis^95^. Attenuated psychotic symptoms typically decline overtime in longitudinal studies and there are CHR individuals who achieve complete remission within the first few months or even weeks^83^, although the differences in those who remit early versus later have not been established.

The outcome trajectories in CHR youth display the same heterogeneity as is found in clinical presentation. The primary outcome focus for CHR individuals has been transition to psychosis, even though fewer than 25% of those who meet established CHR criteria will go on to develop a psychotic illness^3^. However, far less is known about the individuals who meet CHR criteria, but will not go on to develop a psychotic illness^3^. Recent research^96^ has suggested that 43–56% of those who do not make the transition to psychosis experience remission of their attenuated psychotic symptoms. Unfortunately, even where there is remission of symptoms, there is still evidence of poor functioning. In fact, when improved functioning was added to symptom remission, less than 40% of those who did not make the transition to psychosis met criteria for being “in remission”^97^. Recent papers from NAPLS^98^ further differentiated those who did not convert to psychosis into three groups based on symptom ratings at 24 months: (1) those in remission defined as having subthreshold ratings on attenuated psychotic symptoms, (2) symptomatic individuals defined as those who continue to have non-worsening attenuated psychotic symptoms and (3) progression where attenuated psychotic symptoms either worsened or new symptoms emerged. Individuals from the three trajectory groups differed in functioning, cognition and a range of symptoms at 24 months.

Recent studies have also provided support for heterogeneous trajectories in CHR outcome^99^. In one study, using latent profile analysis, three separate classes of at-risk individuals emerged^100^. Class 1 were considered “mild” with the lowest transition rate at 5.6%, had low scores on attenuated psychotic symptoms, depression and intact neurocognition. Class 2 were “paranoid-affective” and had high levels of suspiciousness, mild negative symptoms, moderate depression and a 14.2% transition rate. Finally, Class 3 was described as “negative-neurocognitive” and had the highest levels of negative symptoms, as well as the greatest level of neurocognitive, social cognitive and functional impairment. Moreover, the rates of conversion to psychosis in Class 3 were 29.3%.

A second very recent study, using group-based multi-trajectory modelling^99^, a novel analysis method, which parses out groupings based on the outcomes, described three distinct profiles that were observed in a large sample of CHR individuals. The first group evidenced rapid symptomatic and functional improvement with 50% having good outcomes in symptoms and functioning; the second group demonstrated moderate improvement across symptom and functioning domains with only 25% reaching favourable outcomes; and in contrast the third group exhibited moderate to severe impairment in symptoms and functioning that persisted and did not reach any remission criteria. Although the profiles vary in these three projects, they all describe three outcome trajectories mild, moderate and severe. These analyses provided further support for heterogeneity in the presentation, symptomatology and outcome found in CHR individuals.

Thus, the need to address heterogeneity in the CHR field has important implications for both assessment and treatment. However, it is important to recognize that a research paradigm for studying CHR heterogeneity has opened new directions for both clinical staging and transdiagnostic models of research^101^.

## Conclusion and next steps

Concentrated efforts have already been made to develop a more complete understanding of those at CHR of developing psychosis, to predict who may go on to develop psychosis, and to intervene to prevent the later occurrence of psychosis. However, as this review has described, there are uncertainties in the management of CHR individuals some of which can be attributed to limited treatments to prevent transition to psychosis, as well as the need for interventions for those who do not make the transition to psychosis yet continue to present with many difficulties. Negative symptoms are a major concern but are rarely addressed, and social functioning, which reportedly has a role in later conversion, has never been specifically addressed. Moreover, there is a lack of specific diagnostic tools that can properly identify CHR individuals and potential subtypes. Although there are promising ongoing efforts using different modalities to develop diagnostic biomarkers, such as neuroimaging, electrophysiology, neurocognition, serology, these are in the early stages.

Thus, what is currently needed are sensitive and specific diagnostic criteria, validation of biomarkers, and proof of effectiveness of both psychological interventions and therapeutic agents^102^. Lieberman and colleagues note that although imaging, electrophysiologic and serologic measures are showing promise as diagnostic markers, they require validation in studies with large CHR samples. The methodology has to be rigorous and reliable enough that it can be applied robustly across multiple sites and eventually implemented and obtained in community settings^102^. With respect to treatment, consideration of the heterogeneity of the clinical high-risk population would lead to specific treatments for specific subgroups being tested with the modality of treatment studies being specifically designed to address the presenting problem.

In addition to the heterogeneity mentioned above, there is retrospective evidence that some individuals may develop a first episode of psychosis without passing through an identifiable CHR period^103,104^. One implication^104^ is that if the majority of CHR youth do not develop a psychotic illness and there are individuals who may develop a psychosis without passing through the CHR stage, then the CHR stage, although the most likely, may not be the only pathway to full-blown psychotic disorders.

As outlined in this review, future CHR research must be collaborative, accessible and harmonized with respect to criteria and outcomes. Unfortunately, there have been critics of the field raising issues that are often unfounded^105–107^ and which have had to be addressed in thorough counter arguments by experts in the field^108^. Thankfully, there are already initiatives underway that have adopted these steps. Improved standardization of ascertainment and assessment of CHR individuals can occur through ongoing research in large consortiums, such as NAPLS^109^, PSYSCAN (https://www.psyscan.eu/), Promoting Resilience Outcome and Novel Integrated Approaches to psychosis and depression (PRONIA) (https://www.pronia.eu/) and The Philadelphia Neurodevelopmental Cohort (PNC)^110^, and can lead to further improvement of prediction in this field. In fact, an ongoing project, the National Institute of Mental Health’s (NIMH) Harmonization of At-Risk Multisite Observational Networks for Youth (HARMONY), which is a consortium of the above consortiums, will allow cross-validation of predictive data analytic methods. Some of the tasks of the HARMONY group will be to develop specific cross-study diagnostic criteria. Experimental medicine studies, the intent of which is to test new entities aimed at specific targets, may be considered for this population. Finding preventive treatments for psychosis is an aim of the NIMH Accelerating Medicine Partnership between industry, investigators and government. Finally, recent funding opportunities from NIMH propose to establish large research networks encompassing many international sites that will rapidly recruit large numbers of CHR individuals in order to dissect the heterogeneity and predict differential outcomes to inform future treatment development, which represent a huge step forward for the field. Incorporating our knowledge from past work into these exciting new opportunities has established a promising future for the CHR field.
